# Assembly of skin microbiomes is more neutral than gut microbiomes in multiple animal species

**DOI:** 10.1128/spectrum.02223-25

**Published:** 2025-10-22

**Authors:** Killian D. Campbell, Brendan J. M. Bohannan, Karen L. Adair

**Affiliations:** 1 Department of Biology, Institute of Ecology and Evolution, University of Oregon 209847, Eugene, Oregon, USA; National Center for Genetic Engineering and Biotechnology, Khlong Luang, Pathum Thani, Thailand

**Keywords:** microbiome, microbial ecology, nonhuman microbiome

## Abstract

The gut and external tissues of most animals are colonized by communities of microorganisms that can influence the health, development, and fitness of the host. The composition of these communities can vary greatly between individuals within a host species, and both selective factors (e.g., host immune response) and neutral processes (e.g., random loss of microbial cells) have been shown to contribute to this variation. Although it is known that microbiome composition differs between tissues within an individual host, less is known about the ecological processes that underlie these differences. To address this, we investigated whether the contribution of neutral ecological processes to microbiome assembly differs between external (skin and scale) and internal (gut) host tissues for a diverse panel of animal hosts. To do this, we fit a neutral ecological model to microbial communities from external and internal tissues across a variety of animal hosts. Strikingly, we discovered that the neutral model was equally or a better fit to skin or scale microbial communities across all hosts, suggesting that neutral processes play a larger role in the assembly of skin or scale microbiomes compared with gut microbiomes. Furthermore, we observed that this trend is robust to different definitions of the metacommunity (i.e., the microbial taxa available to colonize a host). Finally, we leveraged a simulation framework to compare the model fits of empirical versus simulated microbial communities. We found that neutral model fits to empirical communities can differ from simulated communities, emphasizing the importance of temporal sampling in profiling animal microbiomes.

**IMPORTANCE:**

Animal microbiomes are complex assemblages of microorganisms that influence a wide variety of host phenotypes. Despite their importance, we lack a thorough understanding of the processes that guide the formation of microbiomes (i.e., microbiome assembly). Understanding how microbiomes assemble is essential to managing microbiomes for host health, conservation, and other goals. Our work highlights the relatively underappreciated role of neutral ecological processes (the random loss or gain of microbial cells) in the assembly of animal microbiomes. We document a potentially general trend: the microbiomes of external tissues (i.e., skin or scales) tend to be more neutrally assembled than those of internal tissues (i.e., guts). This observation suggests that the commonly reported differences in microbiome composition of external and internal animal tissues may be due in part to different assembly processes. Our work also highlights the dynamic nature of microbiomes and the importance of longitudinal sampling when studying animal microbiomes.

## OBSERVATION

Most animals are colonized by communities of microorganisms that influence their development, health, and fitness (1, 2). In healthy individuals, these host-associated microbial communities, or microbiomes, are predominantly found in the gastrointestinal tract, respiratory system, and external tissues (e.g., skin or scales). It is clear that microbiome composition is strongly influenced by body site (3–6), but the ecological processes that underlie this variation remain unclear.

Two main types of processes contribute to the assembly of a microbiome over relatively short time scales. Selective processes are the result of fitness differences among members of a community. Examples of selective factors include variation in oxygen tolerance, ability to grow on specific carbon sources, and competitive or mutualistic interactions (1, 2). In contrast, neutral processes are selectively neutral with respect to fitness and instead are the result of purely stochastic events. Examples of neutral processes include ecological drift (random birth and death of individuals in a community) and passive dispersal of individuals (e.g., random sampling of individual colonists from a source pool) (7, 8). Understanding the relative roles of selective and neutral processes in microbiome assembly is essential to managing microbiomes for host health, conservation, and other goals (8).

Here, we investigate whether the contributions of selective and neutral processes differ between external (e.g., skin or scales) and internal (i.e., gastrointestinal tract) host tissues, and whether these patterns are consistent across host taxa. We identified 16 published data sets that used 16S rRNA gene amplicon sequencing to characterize both the external and gut microbiomes of at least 20 individuals in a set of diverse animal hosts, including humans (Table S1). For each study, the raw sequencing reads were acquired as fastq files from the NCBI Sequence Read Archive or other publicly available database and processed with the Qiime2 platform v2020.2 (9). Amplicon sequence variants (ASVs) were resolved with the dada2 pipeline (10) and classified with a naïve Bayes classifier pre-trained on the Silva 138 SSU database (11–13). Reads for all individuals were rarefied to a uniform read depth matching the lowest read depth from a given individual in the study using the vegan package in R (14). To determine the relative contribution of neutral processes to microbiome assembly, we assessed the fit of the Sloan Neutral Community Model for Prokaryotes (SNCM) (15) to the distribution of microbial taxa in these data sets as previously described (8).

Applying a metacommunity theoretical framework, the SNCM predicts the relationship between the proportion of host individuals (local communities) in which a microbial taxon occurs and its mean relative abundance across all hosts (the metacommunity). In this model, which assumes equal rates of per-capita growth and death, taxa that are only present in a few hosts are likely to be lost due to ecological drift, whereas abundant taxa are most likely to spread among hosts due to passive dispersal. The model is fit to the observed data with the single free parameter *m*, the probability that a random loss will be replaced by reproduction from the same microbial community versus dispersal from another host (8, 16). Microbial taxa that fell within a 95% CI around the model prediction were considered to follow neutral assembly processes as described previously (8).

**Fig 1:**
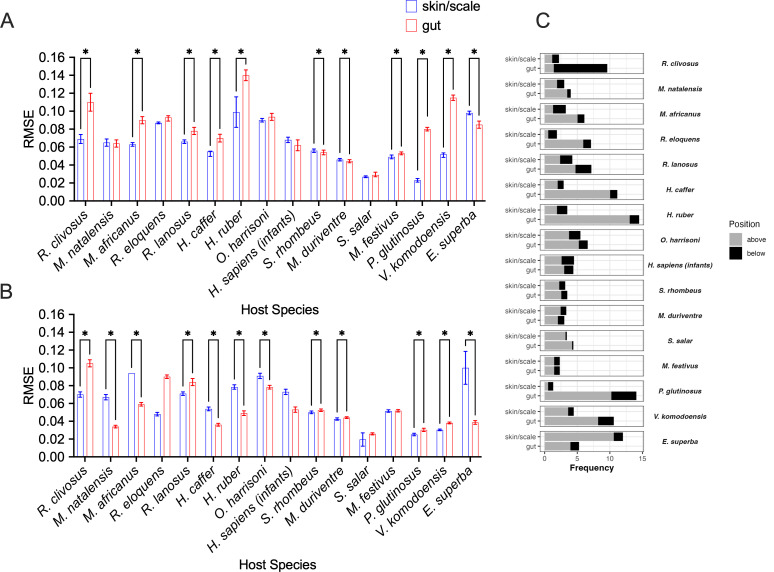
(**A**) Neutral model fits of empirical microbial communities for both skin/scale and gut microbial communities across animal hosts. (**B**) Neutral model fits of simulated microbial communities for both skin and scale and gut microbial communities based on community metrics obtained from corresponding empirical data. Error bars for both A and B represent 95% confidence intervals based on bootstrapping of data. (**C**) Proportion of microbial taxa that fell outside of neutral model predictions for skin/scale and gut communities. Asterisks denote where significance of *P* < 0.05 was detected with a paired *t*-test.

We first compared the fit of the neutral model for microbial taxa (ASVs) in gut and skin or scale tissues separately for each host species, with the metacommunity restricted to samples of the same tissue type. Model fit was assessed by the root mean squared error (RMSE) metric, with lesser RMSE values indicating a better fit of the SNCM to the data. Across nearly all animal hosts, skin or scale microbiomes were equally or significantly better fit to the neutral model compared with gut microbiomes (Fig. 1A). Only one species, *Euphasia superba* (Antarctic krill), departed from this trend, indicating perhaps a unique selective environment of the skin/scale microbiome of this species. These results suggest that overall, the assembly of microbiomes associated with skin or scale tissues tends to be more governed by neutral processes than gut microbiomes. However, the strength of this pattern varied among host taxonomic groups; for example, the fit of the neutral model did not differ between skin or scale and gut tissues for the fish data sets considered in this study, whereas skin microbiomes tended to be a better fit to the neutral model than gut microbiomes for mammal hosts.

Next, we sought to compare the neutral model fits of empirical communities with simulated microbial communities (Fig. 1B). Simulating microbial communities allowed us to obtain a steady-state level of neutrality against which we could compare our empirical communities. This is crucial, as neutrality levels may vary over time. For example, variations in neutrality are sensitive to “selective” factors that may be present at varying intensities at different points in time, including changes in host immunological state due to immune system development or pathogen exposure (17, 18). We leveraged an existing simulation (16) framework to generate completely neutral communities that had the same number of hosts, microbial diversity, and migration parameter “m” as the model fits from each empirical data set. Simulating communities recapitulated the trends in neutrality observed in many of the skin communities (Fig. 1B). However, this trend was not as universal as what was observed in the empirical data. Specifically, there were some instances of gut microbiomes being more neutrally assembled than skin/scale microbiomes in the simulated data, indicating the potential possibility for contributions of neutrality to change over time (Fig. 1B). Moreover, the model was not uniformly better fit to simulated communities, indicating that microbial community composition can vary over time and that longitudinal sampling may be necessary to fully understand microbiome assembly (Fig. 1A and B).

To better understand the factors that contribute to the observed patterns of neutrality in skin/scale versus gut communities, we looked for patterns in the percentage of microbial taxa that fell outside of neutral model predictions across the different hosts for each tissue type. Taxa that occur more or less frequently than neutral model predictions are thought to be non-neutrally distributed and therefore may be subject to selective processes (Fig. 1C). For example, taxa that occur in more individual hosts than predicted by the neutral model (above the neutral model prediction) may be adapted to persist in the host environment or selected for by the host. In contrast, taxa below the neutral model prediction (i.e., detected in fewer samples than predicted by the SNCM) could be particularly dispersal-limited or may be selected against by the host immune system. For nearly all the data sets considered in this study, the majority of the “non-neutral” ASVs fell into the “above partition,” suggesting that these taxa are particularly host-adapted (Fig. 1C). For the few host species where the SNCM was a better fit for the gut microbiome than the skin or scale microbiome, this was also primarily due to a higher proportion of taxa in the above partition, suggesting that for a minority of host species, the gut environment is favorable to more microbial taxa than the skin or scale environment.

**Fig 2:**
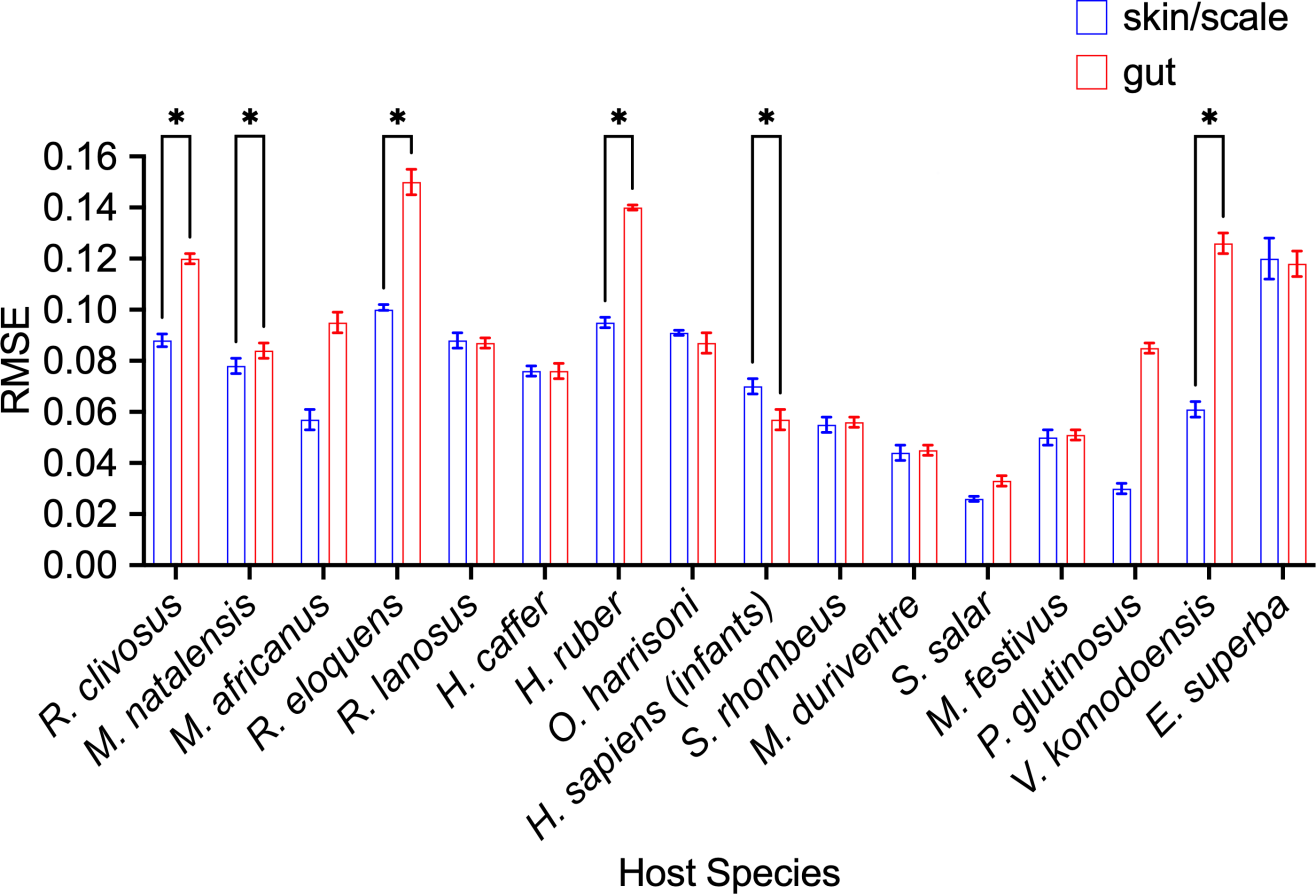
Neutral model fits of empirical microbial communities with the source pool of microbes defined to contain microbes from both tissue types. Error bars represent 95% confidence intervals generated by bootstrapping data. Asterisks denote where significance of *P* < 0.05 was detected with a paired *t*-test.

Finally, we assessed whether changing the definition of the metacommunity to include microbes from both the skin and scale tissues and the gut tissues of a given species influenced the fit of the neutral model. Microbes have been shown to disperse among body sites within a host individual, which suggests that a less tissue-specific definition of the metacommunity may be more appropriate (19–21). Overall, the observation that assembly of gut microbiomes tended to be equally or less neutral than the assembly of microbiomes in skin or scale tissues is consistent whether the metacommunity is restricted to a particular tissue type or includes both tissue types (Fig. 2). However, there were some exceptions (e.g., *E. superba*), suggesting that careful consideration of the source and sink communities is essential for an accurate assessment of the contribution of neutral process to the assembly of host-associated microbiomes.

In this study, we used a modeling-based approach to understand the ecological processes that underlie the assembly of microbiomes associated with gut and skin or scale tissues across a panel of animal hosts. This approach revealed that for the majority of animal species considered, the skin or scale microbiome was more neutrally assembled than the gut microbiome. This may be the result of variation among body sites in susceptibility to microbes dispersing from the environment or host activities that promote/deter microorganisms. We also noted variation among broad host taxonomic groups in the contribution of neutral processes to microbiome assembly among tissue types. Determining the factors that drive these differences (e.g., host lifestyle or immune system complexity) will be an important avenue of future research. Overall, our observations suggest that the gut environment tends to be more selective than the skin or scale environment and showcase the utility of this method to reveal potentially unique features of tissue environments in animal hosts.

## SUPPLEMENTAL MATERIAL

Table S1 spectrum.02223-25-s0001.docx Published data used in this study.

ASM does not own the copyrights to Supplemental Material that may be linked to, or accessed through, an article. The authors have granted ASM a non-exclusive, world-wide license to publish the Supplemental Material files. Please contact the corresponding author directly for reuse.
